# Burden of hidden migraine among the Arab general population: a cross-sectional study

**DOI:** 10.1186/s10194-025-01974-9

**Published:** 2025-02-27

**Authors:** Mohamed A. Elzayat, Shorouq A. Kassab, Mona A. F. Nada, Abdel-Hady El-Gilany

**Affiliations:** 1 Faculty of Medicine, Mansoura University, Mansoura, Egypt; 2 Neutromedica Research Team, Mansoura, Egypt; 3 Department of Neurology, Cairo University, Giza, Egypt; 4 Public Health and Community Medicine Department, Faculty of Medicine, Mansoura University, Mansoura, Egypt

**Keywords:** Arab, Disability, Migraine, Psychological distress, Underdiagnosis

## Abstract

**Background:**

Migraine is a common type of primary headache which is responsible for one-third of the headache cases. It’s also considered the third highest neurological disease with disability in 2021, however, underdiagnosis of migraine remains a significant health problem. This study aims to assess the prevalence of hidden migraine identified by screening among the Arab general population, describe the characteristics of headache attacks, and assess disability and distress associated with migraine.

**Methods:**

This cross-sectional study was conducted between April and June 2024 among the general population of eight Arab countries using a self-administered online questionnaire to collect sociodemographic data and medical history. The questionnaire also included the ten-item Kessler Psychological Distress Scale (K10), Migraine Screen Questionnaire (MS-Q), and Migraine Disability Assessment Questionnaire (MIDAS).

**Results:**

A total of 2152 individuals completed the questionnaire with a median age of 24 (21–29). Among them 683 (31.7%) individuals were screened positive by MS-Q. Using regression analysis, the independent predictors for positive screening were being Saudi Arabian, having one or more diseases, and having severe psychological distress with adjusted odds ratios of 0.622, 0.282, and 1.329 respectively. Among positive cases, 667 (97.7%) reported having headaches in the past 3 months. Phonophobia (50.97%) and photophobia (49.33%) were the most common associated symptoms. Sleep disturbance (66.72%) and noise (63.87%) were the most common triggering factors while sleep (71.81%) and self-medication (68.52%) were the most common relieving factors. Only 25.34% reported having aura with the last attack. According to MIDAS scores, 459 (67.2%) positive cases had moderate or severe disabilities. Regression analysis identified being a housewife and having one or more diseases as the independent predictors of having moderate or severe disabilities with adjusted odds ratios of 0.228 and 0.523 respectively.

**Conclusion:**

Migraine is still underdiagnosed in Arab countries which causes significant disability among positive cases. Raising awareness about the importance of early migraine diagnosis is crucial for encouraging the general population to seek medical advice once they have symptoms.

## Background

Migraine is a common neurological condition that affects over one billion people worldwide [1]. According to the Global Burden of Disease Study 2021 (GBD2021), migraine was considered the third highest neurological condition with age-standardised disability-adjusted life-years (DALYs), the leading cause of DALYs among older children and adolescents and the second leading cause in adults, with a higher prevalence in females [1]. Migraine impact extends beyond individuals directly affected; it also influences their families, colleagues, employers, and society [2]. In addition to significantly impairing the patient’s quality of life and ability to work, migraine also generates a large economic burden on society [3].

Unfortunately, underdiagnosis remains a significant public health issue affecting various conditions including migraine [4]. Despite its considerable burden, migraine continues to be underestimated, under-recognized, underdiagnosed, and under-treated. Globally, the challenges responsible for inadequate migraine care vary but can be categorised into three major categories: social (e.g., Myths regarding headaches), clinical (e.g., wrong diagnosis, treatment, or referral), and financial and political (e.g., poor compliance and low emphasis on headaches) [5]. In addition to that, a definitive diagnostic test for migraine does not exist as it mainly depends on clinical diagnosis, and many cerebral disorders may cause migraine-like symptoms such as infectious, vascular, or neoplastic intracranial lesions [6].

Several studies showed that most migraineurs don’t tend to seek medical advice, and even fewer are correctly diagnosed or get the guideline-recommended therapies [7–9]. However, they should consult an experienced healthcare professional in migraine management, get a correct diagnosis, and receive a personalized treatment that adheres to the recommended guidelines [6]. Results from the study done by Pascual and colleagues (2023) in Spain revealed that approximately 30% of migraineurs hesitated to seek medical care, most thought it was not a serious condition, and only 56.2% of them were accurately diagnosed with migraine and the remaining were undiagnosed even though they met the International Classification of Headache Disorders, 3rd edition (ICHD-III) criteria for migraine [10].

Migraine underdiagnosis is still a significant health problem in different regions of the world, including the Arab world and as far as we know, no studies have measured the magnitude of this problem in Arab countries. So, our aim was to assess the prevalence of hidden migraines among the general population in Arab countries and associated disability and psychological distress to help in lifting this burden in the Arab world.

## Methods

### Study settings

A cross-sectional study with an analytical component was conducted from April 2024 to June 2024 among the general population of eight Arab countries (Egypt, Libya, Algeria, Sudan, Jordan, Saudi Arabia, Yemen and Iraq). These countries were selected to cover different geographic areas considering the availability of local collaborators for data collection.

### Target population

The target population was adults (18 years or older) who hadn’t been diagnosed with migraine in the general population.

### Sample size

The sample size was calculated using Openepi online program (https://www.openepi.com) based on the pooled proportion of migraine in Saudi Arabia (22.56%) reported in a previous systematic review [11] with an α error = 0.05, β error = 0.10 and confidence limit (precision) of 0.05. The calculated sample size was 269 which was multiplied by eight due to design effect, so the final estimated sample size was 2152.

### Sampling technique and data collection approach

The sample size was distributed proportionally according to the population count of each country. The population count was provided from the World Bank Group website [12] or the last census of the country [13].

The questionnaire was distributed as a Google Form. At least one collaborator from each country who was either a medical student or a healthcare worker, and a member of the Neutromedica Research Team, was responsible for distributing the questionnaire links using all social media platforms to collect the required sample.

### Instrument

A self-administered Arabic online questionnaire, which was designed by the investigators according to available Arabic tools in the literature, was used to collect data from the selected population and included the following:

- Section 1: Sociodemographic data, family history of migraine, co-morbidities and smoking status.
- Section 2: The Kessler Psychological Distress Scale (K10) Arabic version was used to measure distress [14]. It is a valid, reliable and easy-to-use tool with a Cronbach alpha coefficient of 0.88. The K10 scale includes 10 questions about emotional states with a response scale from one (none of the time) to five (all the time). The K10 scores lie between 10 and 50 and are categorized as: 10–19 Likely to be well, 20–24 Likely to have a mild disorder, 25–29 Likely to have a moderate disorder, 30–50 Likely to have a severe disorder [15].
- Section 3: The Arabic version of Migraine Screen Questionnaire (MS-Q) which is a simple, valid and reliable tool to screen for migraine with a satisfactory Cronbach alpha coefficient. This questionnaire consists of five questions about the frequency and characteristics of headaches, as well as the presence or absence of migraine symptoms. A score of 0 is obtained for each negative answer (no), and 1 for each positive answer reply (yes). A cutoff point indicating suspicion of migraine is established at 4 points, while a score less than 4 indicates no suspicion of migraine [16].
- Section 4: This section included questions about migraine attacks in the past three months and detailed questions about the last migraine attack. To avoid recall bias, only respondents who had headache attacks in the past three months answered this section. Questions included: frequency, triggering factors, relieving factors and associated symptoms with headache attacks in the past 3 months. In addition, detailed questions related to the last attack duration, intensity, character, site, and presence of aura.
- Section 5: Included the Arabic version of Migraine Disability Assessment Questionnaire (MIDAS) to assess the degree of disability [17]. The questionnaire consisted of five questions. The total number of days is summed as MIDAS score and categorized as follows: grade I (0–5) has little or no disability, grade II (6–10) has mild disability, grade III (11–20) has moderate disability, grade IV (21 or more) has severe disability.

### Statistical analysis

The collected data was coded, processed, and analyzed using Statistical Package for Social Studies (SPSS) (version 29). The appropriate statistical test was used according to the data analyzed when needed. P values at 0.05 were considered statistically significant.

Chi-square was used to test the significance of categorical data. Crude Odds Ratio (COR) and their 95% Confidence Interval (CI) will be calculated. Associations with a p-value of less than 0.2 in the univariate analysis were entered into the logistic regression model to avoid overfitting of the model and the Adjusted Odds Ratio (AOR) and their 95% Confidence Interval (CI) were calculated to predict the independent variables.

## Results

A total of 2152 individuals completed the questionnaire. Most of them were females (1380 (64.1%)) and the majority were living in urban areas (1619 (75.2%)) and were single (1624 (75.5%)). Full characteristics are presented in Table 1. From the total participants, 683 (31.7%) were screened positive for migraine by MS-Q, with statistically significant differences in sex, having one or more diseases, and K10 categories. Logistic regression analysis identified being Saudi Arabian, having one or more diseases, and having severe psychological distress as the independent predictors for positive screening with adjusted odds ratios of 0.622, 0.282 and 1.329 respectively (Table 2).

**Table 1:** Overall prevalence of suspected migraine and its variation according to socioeconomic factors and medical history

Variable		Total Participants *n* (column%)	Positive cases *n* (row%)	Chi-square, *P*-value
Overall		2152 (100)	683 (31.7)	
Age	18–24	1170 (54.4)	384 (32.8)	5.162, 0.076
	25–54	916 (42.6)	286 (31.2)	
	55 and more	66 (3.1)	13 (19.7)	
Sex	Female	1380 (64.1)	416 (30.1)	4.506, **0.034**
	Male	772 (35.9)	267 (34.6)	
Nationality	Egyptian	732 (34)	227 (31)	10.492, 0.162
	Sudanese	321 (14.9)	109 (34)	
	Algerian	311 (14.5)	103 (33.1)	
	Iraqi	297 (13.8)	105 (35.4)	
	Yemeni	230 (10.7)	76 (33)	
	Saudi Arabian	129 (6)	31 (24)	
	Jordanian	80 (3.7)	21 (26.3)	
	Libyan	52 (2.4)	11 (21.1)	
Residence	Urban	1619 (75.2)	507 (31.3)	0.538, 0.463
	Rural	533 (24.8)	176 (33)	
Marital status	Single	1624 (75.5)	530 (32.6)	2.464, 0.292
	Married	493 (22.9)	143 (29)	
	Other	35 (1.6)	10 (28.6)	
Highest educational level	Below secondary school	65 (3)	25 (38.5)	1.572, 0.814
	Secondary school	896 (41.6)	279 (31.1)	
	Bachelor’s degree	926 (43)	293 (31.6)	
	Master’s degree	200 (9.3)	65 (32.5)	
	Doctoral/ fellowship	65 (3)	21 (32.3)	
Employment status	Student	1164 (54.1)	383 (32.9)	2.857, 0.827
	Fulltime employer	514 (23.9)	154 (30)	
	Unemployed	157 (7.3)	52 (33.1)	
	Parttime employer	121 (5.6)	37 (30.6)	
	Self-employed	85 (3.9)	25 (29.4)	
	Housewife	85 (3.9)	26 (30.6)	
	Other	26 (1.2)	6 (23.1)	
Family income	Not enough	207 (9.6)	61 (29.5)	0.603, 0.740
	Enough without reserve	1227 (57)	390 (31.8)	
	Enough with reserve	718 (33.4)	232 (32.3)	
Smoking	Never smoker	1866 (86.7)	584 (31.3)	2.630, 0.452
	Former smoker	69 (3.2)	24 (34.8)	
	Current some day smoker	130 (6)	41 (31.5)	
	Current every day smoker	87 (4)	34 (39.1)	
Family history of migraine	No	1205 (56)	396 (32.9)	1.823, 0.402
	Yes	539 (25)	160 (29.7)	
	Don’t know	408 (19)	127 (31.1)	
Sick with one or more diseases^a^	No	1501 (69.7)	585 (39)	119.920, **< 0.001**
	Yes	651 (30.3)	98 (15.1)	
K10 score	Well	678 (31.5)	186 (27.4)	12.625, **0.006**
	Mild disorder	472 (21.9)	152 (32.2)	
	Moderate disorder	393 (18.3)	122 (31)	
	Severe disorder	609 (28.3)	223 (36.6)	

^a^ Diseases include hypertension, autoimmune diseases, chronic infections, psychiatric diseases, seizures and others

**Table 2:** Predictors of positive screening by MS-Q

Variable		Univariate analysis COR (95% CI)	*p*-value	Multivariate analysis AOR (95% CI)	*p*-value
Age	18–24	Ref		Ref	
	25–54	0.929 (0.772–1.119)	0.438	0.960 (0.755–1.221)	0.740
	55 and more	0.502 (0.270–0.932)	**0.029**	0.599 (0.296–1.212)	0.154
Sex	Female	Ref	**0.034**	Ref	0.271
	Male	1.225 (1.016–1.478)		1.129 (0.910–1.402)	
Nationality	Egyptian	Ref		Ref	
	Sudanese	1.144 (0.865–1.512)	0.345	1.078 (0.797–1.458)	0.625
	Algerian	1.102 (0.830–1.463)	0.503	1.022 (0.758–1.378)	0.886
	Iraqi	1.217 (0.915–1.618)	0.177	1.264 (0.935–1.708)	0.127
	Yemeni	1.098 (0.800-1.506)	0.563	1.091 (0.777–1.533)	0.614
	Saudi Arabian	0.704 (0.456–1.085)	**0.112**	0.622 (0.393–0.985)	**0.043**
	Jordanian	0.792 (0.470–1.335)	0.381	0.681 (0.395–1.175)	0.168
	Libyan	0.597 (0.301–1.183)	**0.139**	0.591 (0.291–1.203)	0.147
Residence	Urban	Ref			
	Rural	1.081 (0.877–1.332)	0.463		
Marital status	Single	Ref		Ref	
	Married	0.843 (0.677–1.051)	**0.130**	0.943 (0.709–1.254)	0.685
	Other	0.826 (0.394–1.732)	0.612	1.052 (0.471–2.350)	0.902
Highest educational level	Below secondary school	Ref			
	Secondary school	0.724 (0.430–1.216)	0.222		
	Bachelor’s degree	0.741 (0.441–1.244)	0.256		
	Master’s degree	0.770 (0.431–1.377)	0.379		
	Doctoral/ fellowship	0.764 (0.371–1.571)	0.464		
Employment status	Student	Ref			
	Fulltime employer	0.872 (0.697–1.092)	0.234		
	Unemployed	1.010 (0.709–1.439)	0.957		
	Parttime employer	0.898 (0.599–1.347)	0.604		
	Housewife	0.899 (0.558–1.448)	0.661		
	Self-employed	0.850 (0.525–1.376)	0.508		
	Other	0.612 (0.244–1.536)	0.295		
Family income	Not enough	Ref			
	Enough without reserve	1.115 (0.808–1.539)	0.507		
	Enough with reserve	1.143 (0.815–1.601)	0.439		
Smoking	Never smoker	Ref		Ref	
	Ex-smoker	1.171 (0.707–1.940)	0.541	1.217 (0.698–2.120)	0.489
	Current occasional	1.011 (0.690–1.483)	0.954	0.952 (0.629–1.441)	0.816
	Current regular	1.408 (0.905–2.190)	**0.129**	1.424 (0.871–2.327)	0.158
Family history of migraine	No	Ref		Ref	
	Yes	0.862 (0.692–1.075)	**0.188**	0.946 (0.751–1.191)	0.635
	Don’t know	0.923 (0.725–1.176)	0.517	1.105 (0.855–1.427)	0.446
Sick with one or more diseases	No	Ref	** < 0.001**	Ref	** < 0.001**
	Yes	0.277 (0.219–0.352)		0.282 (0.221–0.360)	
K10 score	Well	ref		Ref	
	Mild disorder	1.256 (0.972–1.624)	**0.081**	1.116 (0.854–1.457)	0.422
	Moderate disorder	1.191 (0.907–1.563)	0.209	1.072 (0.807–1.424)	0.631
	Severe disorder	1.528 (1.207–1.935)	** < 0.001**	1.329 (1.039-1.700)	**0.023**

Among those who were screened positive for migraine, 667 (97.7%) reported having headaches in the past 3 months. Most cases (60.72%) reported having less than 10 attacks (Low Episodic migraine) in that period. In association with headache, 50.97% had phonophobia while 49.33% had photophobia. Sleep disturbance (66.72%) and noise (63.87%) were the most common triggering factors. In contrast, sleep (71.81%) and self-medication (68.52%) were the most common relieving factors. Regarding the last headache attack, most cases had pressing pain (35.68%), without aura (71.36%), and lasting for one to four hours (35.83%). Full characteristics of migraine attacks affecting those who had headaches in the past 3 months are presented in Table 3.

**Table 3:** Characteristics of migraine attacks

Character		*N* = 667 (%)
Number Of Headache Attacks In The Last 3 Months	Less Than 10	405 (60.72)
	10 To 19	69 (10.34)
	20 To 29	25 (3.75)
	30 Or More	40 (6.00)
	Missing	128 (19.19)
Symptoms Associated With Headache Attacks^a^	Phonophobia	340 (50.97)
	Photophobia	329 (49.33)
	Nausea	179 (26.84)
	Lacrimation	133 (19.94)
	Red Eye	108 (16.19)
	Rhinorrhea	94 (14.09)
	Ptosis	74 (11.09)
	Vomiting	62 (9.30)
	Missing	101 (15.14)
Triggering Factors^a^	Sleep Disturbance	445 (66.72)
	Noise	426 (63.87)
	Anxiety	385 (57.72)
	Sun Exposure	316 (47.38)
	Annoyance	287 (43.03)
	Menstruation	282 (42.28)
	Excessive Heat Exposure	251 (37.63)
	Excessive Light	245 (36.73)
	Emotional Upset	182 (27.29)
	Eating Habits	117 (17.54)
	Physical Activity	102 (15.29)
	Missing	11 (1.65)
Relieving Factors^a^	Sleep	479 (71.81)
	Self-medication	457 (68.52)
	Rest	327 (49.03)
	Hydration	168 (25.19)
	Head Band	105 (15.74)
	Cool Compress Or Ice Pack	71 (10.64)
	Warm Compress	27 (4.05)
	Missing	16 (2.40)
Duration of the last attack	Less than an hour	168 (25.19)
	1–4 h	239 (35.83)
	4–12 h	96 (14.39)
	12–24 h	44 (6.60)
	24 h or more	101 (15.14)
	Missing	19 (2.85)
Severity of the last attack (self-reported)	Mild	134 (20.09)
	Moderate	398 (59.67)
	Severe	120 (17.99)
	Missing	15 (2.25)
Character of last attack pain	Pressing	238 (35.68)
	Throbbing or pulsating	203 (30.43)
	Dull aching	171 (25.64)
	Piercing or drilling	39 (5.85)
	Missing	16 (2.40)
location of the last attack	Bilateral or diffuse	188 (28.19)
	Unilateral	154 (23.09)
	Forehead	143 (21.44)
	Retroorbital	95 (14.24)
	Occipital area	72 (10.79)
	Missing	15 (2.25)
Associated aura with the last attack	No	476 (71.36)
	Yes	169 (25.34)
	Missing	22 (3.30)

^a^ Categories are not mutually exclusive

Positive cases exhibited significant disability according to MIDAS scores with a median score of 18 (interquartile range: 8, 35), with 459 (67.2%) individuals experiencing moderate or severe disability. There were statistically significant differences in employment status and the presence of one or more diseases between positive cases with no or mild disability and those with moderate or severe disability (Table 4). Logistic regression analysis identified being a housewife and having one or more diseases as the independent predictors of having moderate or severe disabilities with adjusted odds ratios of 0.228 and 0.523 respectively (Table 5).

**Table 4:** Migraine disability grades according to MIDAS

Variable		Grades I & II *n* (column%)	Grades III & IV *n* (column%)	Chi-square, *P*-value
Overall		224 (32.8)	459 (67.2)	
Age	18–24	119 (31)	265 (69)	2.193, 0.334
	25–54	102 (35.7)	184 (64.3)	
	55 and more	3 (23.1)	10 (76.9)	
Sex	Female	146 (35.1)	270 (64.9)	2.553, 0.110
	Male	78 (29.2)	189 (70.8)	
Nationality	Egyptian	78 (34.4)	149 (65.6)	2.411, 0.934
	Sudanese	33 (30.3)	76 (69.7)	
	Algerian	31 (30.1)	72 (69.9)	
	Iraqi	33 (31.4)	72 (68.6)	
	Yemeni	29 (38.2)	47 (61.8)	
	Saudi Arabian	11 (35.5)	20 (64.5)	
	Jordanian	6 (28.6)	15 (71.4)	
	Libyan	3 (27.3)	8 (72.7)	
Residence	Urban	172 (33.9)	335 (66.1)	1.137, 0.286
	Rural	52 (29.5)	124 (70.5)	
Marital status	Single	167 (31.5)	363 (68.5)	2.567, 0.227
	Married	52 (36.4)	91 (63.6)	
	Other	5 (50)	5 (50)	
Highest educational level	Below secondary school	7 (28)	18 (72)	4.572, 0.334
	Secondary school	84 (30.1)	195 (69.9)	
	Bachelor’s degree	101 (34.5)	192 (65.5)	
	Master’s degree	27 (41.5)	38 (58.5)	
	Doctoral/ fellowship	5 (23.8)	16 (76.2)	
Employment status	Student	116 (30.3)	267 (69.7)	13.000, **0.043**
	Fulltime employer	50 (32.5)	104 (67.5)	
	Unemployed	20 (38.5)	32 (61.5)	
	Parttime employer	14 (37.8)	23 (62.2)	
	Housewife	16 (61.5)	10 (38.5)	
	Self-employed	7 (28.0)	18 (72.0)	
	Other	1 (16.7)	5 (83.3)	
Family income	Not enough	21 (34.4)	40 (65.6)	0.417, 0.812
	Enough without reserve	124 (31.8)	266 (68.2)	
	Enough with reserve	79 (34.1)	153 (65.9)	
Smoking	Never smoker	190 (32.5)	394 (67.5)	0.296, 0.961
	Former smoker	9 (37.5)	15 (62.5)	
	Current some day smoker	14 (34.1)	27 (65.9)	
	Current every day smoker	11 (32.4)	23 (67.6)	
Family history of migraine	No	127 (32.1)	269 (67.9)	0.502, 0.778
	Yes	52 (32.5)	108 (67.5)	
	Don’t know	45 (35.4)	82 (64.6)	
Sick with one or more diseases	No	180 (30.8)	405 (69.2)	7.602, **0.006**
	Yes	44 (44.9)	54 (55.1)	
K10 score	Well	60 (32.3)	126 (67.7)	4.784, 0.118
	Mild disorder	53 (34.9)	99 (65.1)	
	Moderate disorder	48 (39.3)	74 (60.7)	
	Severe disorder	63 (28.3)	160 (71.7)	

**Table 5:** Predictors of moderate or severe disability according to MIDAS score

Variable		Univariate analysis COR (95% CI)	*p*-value	Multivariate analysis AOR (95% CI)	*p*-value
Age	18–24	Ref	-		
	25–54	0.810 (0.586–1.121)	0.203		
	55 and more	1.497 (0.405–5.538)	0.546		
Sex	Female	Ref	**0.110**	Ref	0.173
	Male	1.310 (0.940–1.826)		1.276 (0.899–1.811)	
Nationality	Egyptian	Ref	-		
	Sudanese	1.206 (0.737–1.972)	0.456		
	Algerian	1.216 (0.736–2.009)	0.446		
	Iraqi	1.142 (0.696–1.873)	0.599		
	Yemeni	0.848 (0.496–1.453)	0.549		
	Saudi Arabian	0.952 (0.434–2.087)	0.902		
	Jordanian	1.309 (0.488–3.507)	0.593		
	Libyan	1.396 (0.360–5.411)	0.629		
Residence	Urban	Ref	-		
	Rural	1.224 (0.844–1.777)	0.287		
Marital status	Single	Ref	-		
	Married	0.805 (0.547–1.185)	0.272		
	Other	0.460 (0.131–1.611)	0.225		
Highest educational level	Below secondary school	Ref	-		
	Secondary school	0.903 (0.363–2.242)	0.826		
	Bachelor’s degree	0.739 (0.299–1.829)	0.513		
	Master’s degree	0.547 (0.201–1.492)	0.239		
	Doctoral/ fellowship	1.244 (0.329–4.708)	0.747		
Employment status	Student	Ref	-	Ref	-
	Fulltime employer	0.904 (0.605–1.350)	0.621	0.912 (0.605–1.377)	0.662
	Unemployed	0.695 (0.382–1.266)	0.235	0.671 (0.366–1.231)	0.197
	Parttime employer	0.714 (0.355–1.436)	0.345	0.688 (0.340–1.396)	0.301
	Housewife	0.272 (0.120–0.616)	**0.002**	0.288 (0.125–0.660)	**0.003**
	Self-employed	1.117 (0.454–2.747)	0.809	1.040 (0.416–2.601)	0.933
	Other	2.172 (0.251–18.800)	0.481	2.029 (0.228–18.083)	0.526
Family income	Not enough	Ref	-		
	Enough without reserve	1.126 (0.637–1.991)	0.683		
	Enough with reserve	1.017 (0.561–1.841)	0.956		
Smoking	Never smoker	Ref	-		
	Ex-smoker	0.804 (0.345–1.870)	0.612		
	Current occasional	0.930 (0.477–1.814)	0.832		
	Current regular	1.008 (0.482–2.111)	0.982		
Family history of migraine	No	Ref	-		
	Yes	0.981 (0.662–1.452)	0.922		
	Don’t know	0.860 (0.565–1.310)	0.483		
Sick with one or more diseases	No	Ref	**0.006**	Ref	**0.004**
	Yes	0.545 (0.353–0.843)		0.523(0.336–0.814)	
K10 score	Well	Ref	-		
	Mild disorder	0.889 (0.565-1.400)	0.613		
	Moderate disorder	0.734 (0.456–1.182)	0.203		
	Severe disorder	1.209 (0.792–1.847)	0.379		

## Discussion

This international multicenter study reveals that migraine is still an underdiagnosed health problem in the Arab world. The prevalence of hidden migraine among the studied sample was 31.7% [683 out of 2152 were screened positive for migraine using the Migraine Screening Questionnaire (MS-Q)]. This result is relatively close to the estimated prevalence of migraine headaches (37.2%) among the population living in Jeddah, however, they used ID migraine questionnaire which hasn’t been translated into Arabic or validated among the Arab population yet. In addition, they didn’t exclude patients diagnosed with migraine which could overestimate the results of the screening [18]. The high prevalence of hidden migraine might be due to many factors including lack of accessible and appropriate health care; delayed or non-healthcare seeking; self-medication and easy accessibility of triptans, ergots and analgesics; lack of awareness about migraine and misdiagnosis by general practitioners. Also, people differ in pain sensitivity and thresholds. A multicenter study conducted in seven countries (Brazil, Italy, Moldovia, Mexico, Argentina, Chile, and Uruguay) among migraineurs, found that only 28% were accurately diagnosed by the general practitioner, and only 28.4% of the patients were aware of having migraine [19].

The logistic regression analysis indicated that having one or more diseases decreased the chance of being screened positive for hidden migraine. This may be due to the tendency of those who have one or more diseases to seek medical advice for such diseases, which might play a significant role in their early diagnosis of migraine, so they are less likely to be included in the study as most of them are likely to be diagnosed during their care for the other diseases. Psychological distress, measured by the Kessler Psychological Distress Scale (K10) Arabic version, was estimated to affect 72.8% of positive cases as 32.7% of positive cases reported having severe psychological distress, while 17.9% and 22.3% experienced moderate and mild distress, respectively. In addition, severe psychological distress was an independent predictor for being positively screened for migraine highlighting the significant association between them. This finding is in line with an existing review article that highlights that anxiety and depression are frequently associated with migraines, adversely affecting disease prognosis, treatment efficacy, and clinical outcomes [20]. Psychiatric disorders, especially anxiety, are frequently associated with migraine and arise due to a patient’s emotional and physical stress from migraine episodes. This association may be due to similar or shared pathogenesis and mechanism (e.g. vascular, nervous, and genetic factors) between the two. Another evidence of this association is the number of clinical features shared by both conditions [21].

Hidden migraines are significantly lower in Saudi Arabia (24%) compared to the other countries included in the study. This could be due to better accessibility and affordability of healthcare services in Saudi Arabia which can facilitate earlier diagnosis and treatment, resulting in fewer missed cases in the community. However, this finding is lower than the results reported in the previously mentioned study conducted in Jeddah [18]. This difference may be due to differences in the screening tool and the inclusion criteria discussed before which can affect the prevalence rates reported. The analysis also revealed that males have a higher prevalence of hidden migraine (34.6%) than females (30.1%), which may be due to the societal misconception among males as they often believe that headache is a trivial issue that they can endure so they hardly complain and seek medical care for their headaches. In contrast, the global prevalence of migraine is higher among females than males, this could affect the physicians’ diagnostic decisions, potentially leading to missing migraine cases in males [1]. According to the univariate regression analysis, positive cases in the 18–24 and 25–54 age groups have a higher prevalence of hidden migraine than those who are older, which aligns with the global prevalence of migraine which reported that most of the migraineurs are within the age group of 15–39 [1]. In addition, older patients are more likely to have other diseases which was found to be an independent predictor for not being screened positive for migraine making the prevalence of hidden migraine lower among them compared to younger age groups.

Among positive cases, the most common triggering factors of headache attacks were sleep disturbance and noise, reported by 66.7% and 63.9% of participants respectively. This finding is in line with migraine triggers as reported by previous systematic reviews that confirm the significant role of sleep disturbance and noise as migraine triggers [22, 23]. In contrast, sleep was the most common relieving factor (71.8%). This highlights the complex relationship between migraine and sleep. The bi-directional relationship between sleep and migraine is an area of active research and many researchers are trying to find shared pathophysiology and potential mechanisms. A published literature review highlighted some possible mechanisms for this complex relationship and found that serotonin and dopamine could be the key to understanding why sleep disturbance can trigger migraine while the glymphatic system can offer a clue to understanding how sleep can relieve migraine [24].

Self-medication was the second most common relieving factor (68.5%) highlighting a major problem of self-medication among positive cases, as many of them resort to painkillers for headache relief instead of seeking medical advice. Similarly, a previous study in Italy reported that 67.2% of migraineurs attending headache centres for the first time used over-the-counter self-medication which is very close to our results [25]. Only 25.34% of positive cases reported experiencing an aura with their last attack which is in line with one notable study conducted in Egypt which found that (34.1%) of migraineurs experienced migraine with aura [26]. This result is also consistent with previous studies conducted among migraine patients that indicate that migraine with aura affects around 15 − 40% of the patients with migraine [26–30]. This low percentage of aura among positive cases may contribute to not seeking medical advice as experiencing an aura is an alarming symptom which could encourage patients to become more concerned and seek medical advice.

The Arabic version of Migraine Disability Assessment Questionnaire (MIDAS) was used to assess the degree of disability among the positive cases and revealed that 82.4% of them suffered from disability, which aligns with the global burden of disease in 2021 indicating that migraine is one of the leading causes of disability worldwide [31]. Furthermore, 15.2% of positive cases experienced mild disability, 23.4% had moderate disability and 43.8% had severe disability. These findings exceed the results of previous studies on migraineurs. For example, an observational study revealed that 15.5%, 17.5%, and 24.5% of migraineurs had mild, moderate, and severe disability while another reported that 18.6%, 17.9%, and 11.3% of migraineurs had mild, moderate, and severe disability respectively [32, 33]. This difference especially in severe disability can be attributed to different study populations, as these studies included migraine patients who received acute and preventive medications which can improve their symptoms and degree of disability. Besides, it’s notable that being a housewife and having one or more diseases reduced the possibility of experiencing moderate and severe disability from migraine. This may be due to the low workload on housewives rather than employed individuals. As well, patients with co-morbidities may experience daily disabilities but believe that it’s due to their co-morbid diseases, not migraine.

### Recommendations

Raising awareness of the general population about migraine disease and the importance of early diagnosis and management is mandatory to reduce the burden of hidden migraine and its associated disability in the Arab world. Future studies should focus on detecting the reasons for migraine underdiagnosis and why positive cases don’t seek medical advice despite their symptoms and disability.

### Strengths

This study is the first of its kind to estimate the prevalence of hidden migraine and associated burden among the Arab general population and showed a major health problem of migraine underdiagnosis in the Arab world and its disabling effects using validated scales.

### Limitations

The study used a self-reporting questionnaire that can be affected by under- or over-estimation. Recall bias may affect the responses. In addition, the data was collected using an online questionnaire, which might affect external validity. We used MIDAS to assess the disability among positive cases by screening while it was validated among confirmed migraine patients, which might affect our results, however, it’s noteworthy that MIDAS is the only available scale for measuring disability in migraine. Moreover, given the cross-sectional nature of the study, it was not feasible to establish causal relationships, despite the potential existence of associations between the variables.

## Conclusion

Migraine is still underdiagnosed in Arab countries and is associated with significant distress and disability among positive cases. In addition, self-medication is very common among positive cases complicating the problem. Raising awareness about the importance of early migraine diagnosis is crucial to encouraging the general population to seek medical advice once they have symptoms.

## Data Availability

The datasets generated and/or analyzed during the current study are available from the corresponding author upon reasonable request.

## Abbreviations

K10: Ten-item Kessler Psychological Distress Scale
MS-Q: Migraine Screen Questionnaire
MIDAS: Migraine Disability Assessment Questionnaire
GBD2021: Global Burden of Disease Study 2021
DALYs: Disability-adjusted life-years
ICHD-III: International Classification of Headache Disorders, 3rd edition
SPSS: Statistical Package for Social Studies
COR: Crude Odds Ratio
CI: Confidence Interval
AOR: Adjusted Odds Ratio
MFM-IRB: Institutional Research Board of Mansoura Faculty of Medicine
